# Development of a new nanosensor for the determination of food coloring Sunset Yellow in powder drinks using L-cysteine coated copper nanoclusters

**DOI:** 10.55730/1300-0527.3654

**Published:** 2024-01-18

**Authors:** Mehmetcan BİLKAY, Büşra KARATAŞ, Hayriye Eda ŞATANA KARA

**Affiliations:** Department of Analytical Chemistry, Faculty of Pharmacy, Gazi University, Ankara, Turkiye

**Keywords:** Sunset yellow, copper nanocluster, fluorescence sensor, food dye

## Abstract

Sunset Yellow (SY), which is an artificial azo dye, is preferable for its high stability and low cost. The determination of SY in foods is extremely important for human health because excessive consumption of SY has harmful effects, such as hyperactivity disorder and cancer. In this method, L-cysteine coated copper nanoclusters (CuNCs) were used as a fluorescence probe. L-cysteine has been used as both a reducing and stabilizing agent. One-step green hydrothermal synthesis of CuNCs was made. L-cysteine-coated CuNCs have been characterized using several of methods. CuNCs quenching mechanism is static and inner filter effect (IFE). The linear range is 0.65–14 μg.ml^−1^ at optimum conditions. LOD and LOQ values were calculated as 0.1 and 0.35 μg.ml^−1^, respectively. The proposed method was used for the determination of SY in different type of powder drinks. The developed nanosensor is environmentally friendly, easy, fast, reproducible, and low cost.

## 1. Introduction

In the food industry, frequently used synthetic dyes have become alternatives to natural dyes with their low cost, impressive stability, and color integrity [1,2]. Sunset yellow (SY), disodium 6-hydroxy-5-[(4-sulfophenyl) azo]-2-naphtalenesulfononate, which is one of the synthetic dyes and contains the azo group, is used in beverages, sugars, pharmaceuticals, and cosmetics [1–3]. High intake of SY causes various harmful effects such as suppression of the immune system, cancer, hyperactivity, liver damage and allergies and is banned in Finland and Norway for these reasons [1,4]. The acceptable daily intake of SY is 4 mg/kg according to the European Food Safety Authority (EFSA) [1]. Therefore, it is very important to develop easy, fast, low-cost, reproducible, and compatible with green chemistry analysis methods for the determination of SY. Various analytical methods have been used for the determination of SY, such as electrochemical methods [1–3], high-performance liquid chromatography (HPLC) [5], spectrophotometry [6], capillary electrophoresis [7], and ELISA [8]. However, these applications may have several disadvantages such as long analysis times, the need for specialized personnel, using of expensive equipment, long sample preparation procedures, and not being suitable for green chemistry [9].

Metal nanoclusters, consisting of a few to hundreds of atoms, have unique properties with their ultrasmall size. Metal nanoclusters have properties similar to molecules, such as HOMO-LUMO transition, magnetism, spectrochemical, and redox properties [9–10]. The most well-known metal nanoclusters are copper nanoclusters (CuNCs), silver nanoclusters (AgNCs), and gold nanoclusters (AuNCs), etc. When comparing CuNCs, AgNCs, and AuNCs, copper nanoclusters are more economical than others [10]. Metal nanoclusters have several advantages including, good photoluminescence, low toxicity, biocompatibility, water solubility, high Stokes shift, high photostability, and catalytic properties, therefore, metal nanoclusters can be an alternative to fluorescent dyes, quantum dots, fluorescent proteins, and organic fluorophores [9–11]. Metal nanoclusters have been used in various fields such as pharmaceutical analysis [10], food analysis [9], bioimaging [12], ions sensing [12], and catalysis [13]. There are various synthesis methods for metal nanoclusters such as microwave [14], sonochemical [15], UV light [16], one-step hydrothermal [9], and electrochemical [17]. HSA [18], BSA [10], amino acids such as cysteine [9], tryptophan [12], peptides such as glutathione [19], polymers such as PEI [20] have been used for the coating agents. In this study, CuNCs have been synthesized using copper salt and L-cysteine as inexpensive precursors, and an inexpensive, fast, simple, repeatable, green chemistry-friendly method has been developed for the determination of SY. Various techniques have been used for the characterization of the synthesized CuNCs. These are UV-Vis spectrophotometer to obtain the absorbance spectrum of the synthesized CuNCs, spectrofluorimeter to measure excitation and emission wavelengths and fluorescence intensity, infrared spectroscopy (FT-IR), X-ray photoelectron spectroscopy XPS to elucidate how CuNCs bind to cysteine, transmission electron microscopy (TEM), zeta potential measurement and dynamic light scattering analyze (DLS) for size characterization. The developed fluorescence probe, whose optimum working parameters were determined, was used for the determination of SY in powder drink. The mechanism of fluorescence quenching events that occur as a result of interaction of CuNCs with SY is due to static quenching and inner filter effect.

## 2. Experimental

### 2.1. Chemicals and materials

L-cysteine hydrochloride monohydrate, copper nitrate, glucose, sucrose, magnesium nitrate, phosphoric acid, ascorbic acid, and citric acid were obtained from Merck (Darmstadt, Germany). Sodium hydroxide, sodium chloride, potassium chloride, and calcium chloride were obtained from Sigma (Germany). Sunset Yellow was obtained from Roha Dye Chem. Pvt. Ltd. (Mumbai, India). Powder drinks were purchased from a supermarket in Ankara, Türkiye. All the other chemicals and solvents were of analytical grade. The stock solutions of SY (2.3 × 10^−3^ mol L^−1^) were prepared in deionized water and kept in the refrigerator. Acetate buffer (0.1 M pH 4.00 and 5.00), phosphate buffer (0.1 M pH 3.00, 6.00, 7.00, 8.00), and borate buffer (0.1 M pH 9.00 and 10.00) were prepared in deionized water. The pH was adjusted with 5 M sodium hydroxide. The deionized water was used for all experiments (18 MΩ.cm).

### 2.2. Instrumentation

XPS analyses were made by using PHI 5000 VersaProbe III multi-technique XPS (ULVAC-PHI, Japan). DLS and Zeta potential analysis were done on a Zetasizer Nano ZS Series, Malvern instrument. FEI Tecnai G2 Spirit Biotwin CTEM was used for TEM image. Perkin Elmer Spectrum 400 FTIR/FTNIR spectrometer was used for IR spectrum. To obtain absorbance spectra and fluorescence spectra, Specord 50 Plus (Analytic Jena, Germany) and Agilent Cary Eclipse spectrofluorometer were used, respectively. In fluorescence measurements, the excitation wavelength and slit width were 375 nm and 10.0 nm, respectively. Mettler-Toledo GmbH pH meter was used for pH measurements. All experiments were performed at room temperature.

### 2.3. Synthesis of L-cysteine-coated copper nanoclusters

The synthesis of L-cysteine-coated copper nanoclusters was carried out according to the synthesis procedure in the literature [9]. Shortly, 5 mL of 0.4 M NaOH was taken in a beaker and 175 mg of L-cysteine was added. Then, 500 μL 1.00 mM of CuNO_3_ solution was added to this mixture drop by drop. This mixture was stirred on a magnetic stirrer at room temperature for 30 min to dissolve all precursors. Then, the obtained solution was heated to 55 °C for 4 h. The synthesized CuNCs were stored in the refrigerator at 4 °C.

### 2.4. Sample preparation

The extraction procedure for SY in powder drinks samples is as follows: 30 mg of powder drinks were weighed precisely, dissolved with deionized water, and completed to 10 mL in the volumetric flask. It was centrifuged to remove the undissolved contents. Fifty μL of clear supernatant were used for further analysis. All experiments were repeated three times.

### 2.5. Interaction between SY and copper nanoclusters

The absorption spectra of SY depended on the media pH. Consequently, the effect of pH was identified in the range of 3–10 by using acetate, phosphate, and borate buffer solutions. Maximum absorption intensity was obtained in pH 9 borate buffer. To understand the interaction between CuNCs and SY, 10 μL of CuNCs solution in 2 mL of borate buffer at pH 9.00 was titrated by successive additions of SY. The excitation wavelength was chosen as 375 nm. The fluorescence intensity of CuNCs was quenched with increasing SY concentration. Obtained fluorescence spectra were recorded in the presence and absence of SY. The quenching of fluorescence intensity was recorded as F_0_/F. Measurements were made three times and the obtained values were used in the calculations.

In order to understand the selectivity of CuNCs to SY, the substances in powder drinks that are potential interferents, namely glucose, sucrose, citric acid, ascorbic acid, tartaric acid, and Na^+^, K^+^, Mg^2+^, Ca^2+^, Cl^−^, SO_4_^2−^ ions were examined. Substances expected to interference (0.39 mM) were prepared 30 times more concentrated than SY (0.013 mM).

## 3. Results and discussion

### 3.1. Characterization of synthesized L-cysteine coated copper nanoclusters

**Figure 1:**
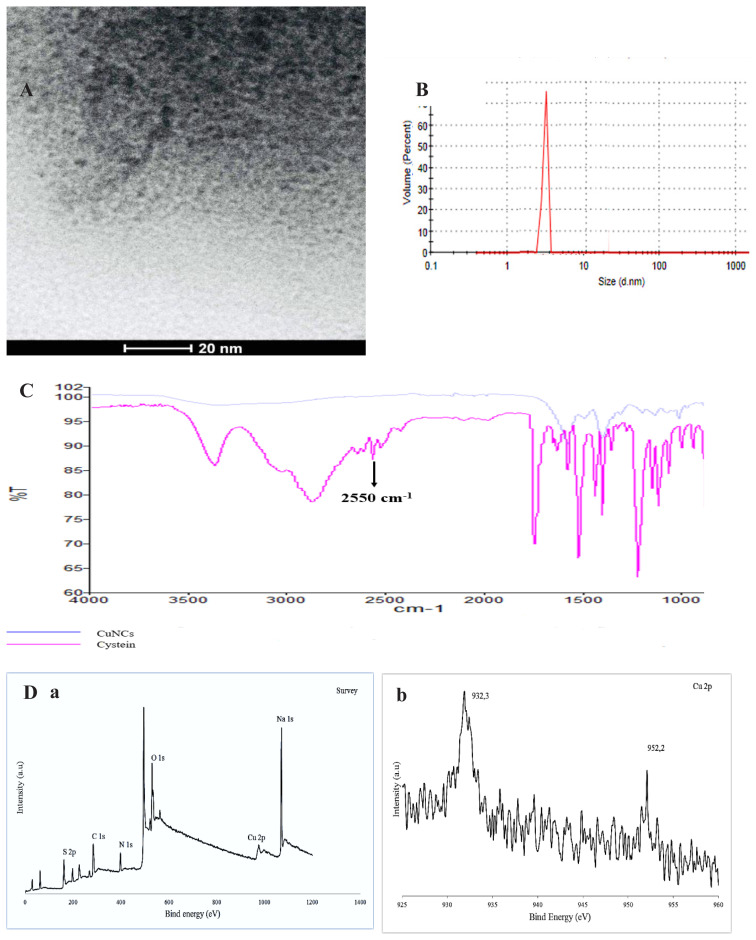
A) TEM image of CuNCs, B) Dynamic light scattering CuNCs, C) FT-IR of L-cysteine (blue line) and CuNCs (pink line) D) a- The survey XPS spectrum of CuNCs and b-XPS spectrum of Cu in CuNCs.

The methods used for the structural and optical characterization of the synthesized CuNCs, were UV-Vis spectrophotometry, spectrofluorimetry, FT-IR, XPS, TEM, DLS, and zeta potential analysis. According to the TEM image, the shape of the CuNCs was found to be spherical, with a size of about 3 nm and a uniform particle size distribution (Figure 1A). DLS analysis results showed that the dimension of CuNCs was 3.2 nm and this result was found to be compatible with the TEM image (Figure 1B). The increase in size resulting from DLS analysis is thought to be due to the hydrodynamic radius. The zeta potential analysis of synthesized CuNCs in its aqueous prepared solution at pH 9 has shown that the nanoclusters have a negative charge (–20.4 mV), and this protects the particles from aggregation.

It was aimed to determine the surface groups of nanoclusters by taking the FT-IR spectra of L-cysteine and CuNCs separately. When the obtained IR spectra were compared, the band corresponding to the -SH stretching vibration in the range of 2550–2600 cm^−1^ disappeared. This result explains that the covalent bond formation between L-cysteine and Cu atoms on the surface of NCs is due to sulfur atoms (Figure 1C) and that the surface of CuNCs is coated with L-cysteine.

The XPS spectrum of synthesized nanoclusters defined that the CuNCs formed of Cu, S, N, C, and O elements. The five peaks belonging to mentioned elements are as follows; 162 eV (S 2p), 285 eV (C 1s), 398 eV (N 1s), 531 eV (O 1s), and 932 eV (Cu 2p). The Cu2p spectrum showed two peaks at 952.2 and 932.2 eV indicating Cu 2p1/2 and 2p3/2, respectively, according well with Cu (0) state. In addition, the absence of a peak around 940 eV explains the lack of Cu^2+^ in the CuNCs and the conversion of all copper ions to metal nanoclusters (Figure 1D).

**Figure 2:**
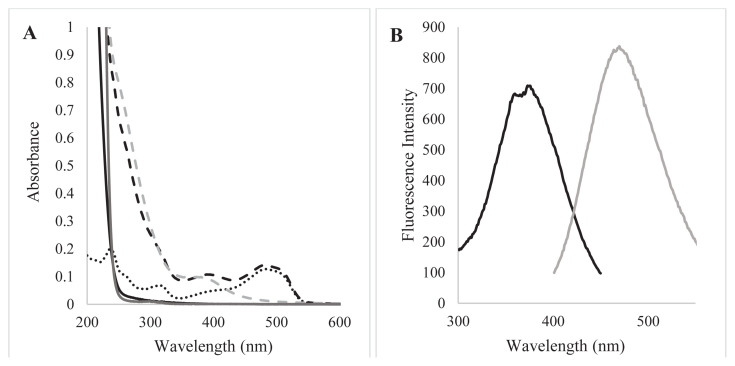
A) Absorption spectra of CuNCs (grey dash line), L-cysteine (black line) and Cu(NO_3_)_2_ (grey line), SY (black dotted line), CuNCs-SY complex (black dash line) B) Excitation (black line), and emission (grey line) spectra of CuNC.

The absorption spectra of Cu(NO_3_)_2_, L-cysteine, and CuNCs are shown in Figure 2A. As can be seen from the figure, CuNCs had a weak absorption peak at 375 nm, while (CuNO_3_)_2_ and L-cysteines did not show a significant peak at 375 nm.

Considering the absorption spectrum of the synthesized CuNCs, it is seen that the maximum peak is at 375 nm. When CuNCs were excited at 375 nm, it was seen that they produced strong blue emission at 470 nm (Figure 2B). When the fluorescence spectra of Cu(NO_3_)_2_, L-cysteine, and CuNCs are compared, it is seen that only CuNCs have a significant emission spectrum. The obtained results indicated the successful preparation of nanoclusters.

As a result, it is understood that the synthesized CuNCs can be used as a fluorescent probe.

### 3.2. Optimization of CuNCs synthesis conditions

**Figure 3:**
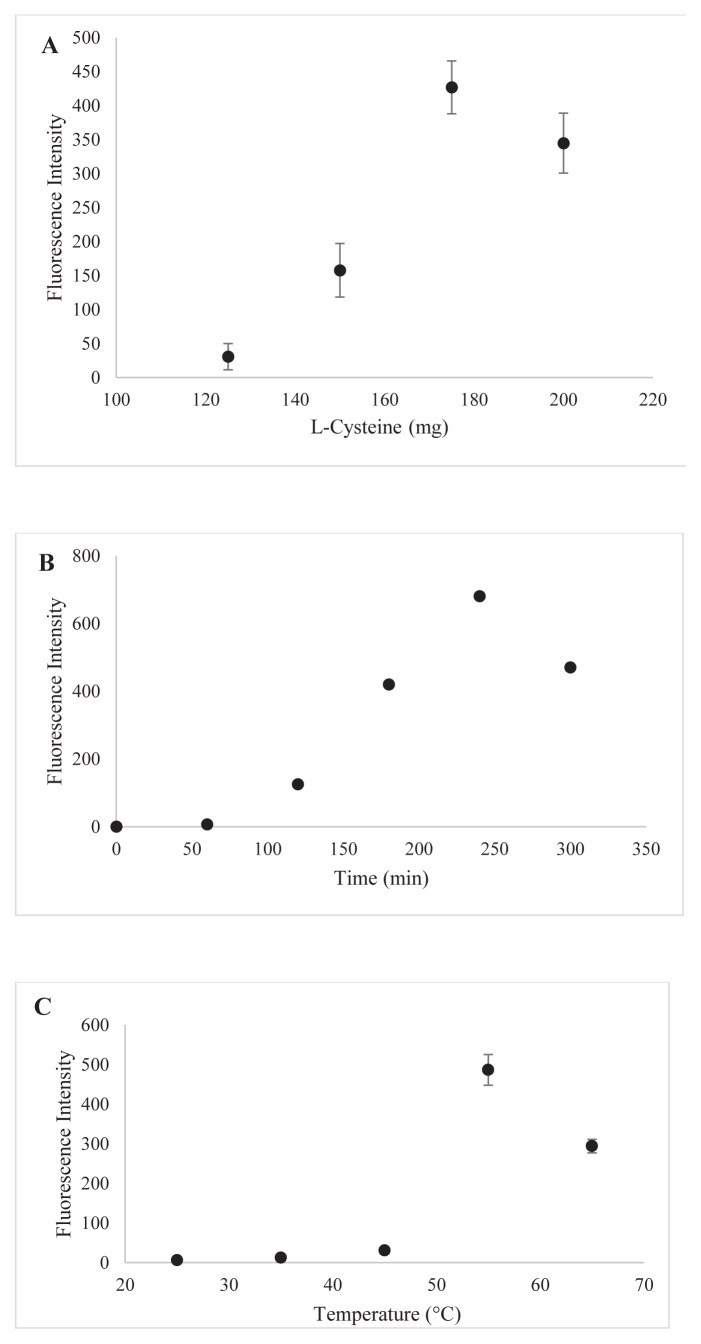
Optimization studies of CuNCs. A) L-cysteine amount, B) time, C) temperature, D) pH.

In this study, L-cysteine has been used as both a reducing and stabilizing agent and the synthesis conditions of CuNCs have been optimized. Reaction time, temperature, copper concentration, and L-cysteine concentrations have been optimized (Figure 3). First, the amount of L-cysteine has been optimized. L-cysteine amounts of 125 mg, 150 mg, 175 mg, and 200 mg have been studied and it was decided that 175 mg of L-cysteine was the optimum condition. Less than 175 mg of L-cysteine was insufficient for the reduction and stabilization of Cu^2+^. The experiment using 200 mg of cysteine has been eliminated because of the occurring precipitation (Figure 3A). For the optimization of the incubation times of CuNCs, measurements were taken at 0 min, 60 min, 120 min, 180 min, 240 min, and 300 min and maximum signal intensity was obtained at 240 min (Figure 3B). The reason for the decrease in fluorescence intensity after 240 min is thought to be due to the aggregate of CuNCs. Indeed, experimental studies have shown that the color of the solution obtained at the end of this period becomes darker and precipitate formation occurs. In the last step of the optimization studies, experiments were carried out at room temperature, 35 °C, 45 °C, 55 °C, and 65 °C to examine the effect of temperature on CuNCs. It is thought that CuNCs are not formed at the desired level, since sufficient energy is not supplied at temperatures below 55 °C (Figure 3C). As a result, 175 mg L-cysteine amount, 240 min incubation time, and 55 °C synthesis temperature were selected as optimized conditions.

### 3.3. The experimental conditions selections

In the study, the change in the fluorescence signal of nanoclusters between pH 3–10 was examined. To examine the effect of pH on CuNCs and Sunset Yellow, pH 4.00, pH 5.00 acetate buffer, pH 3.00, pH 6.00, pH 7.00, pH 8.00 phosphate buffer, pH 9.00, pH 10.00 borate buffer were prepared. There was an increase in the fluorescence signal up to pH 9, but the emission value decreased after this value. CuNCs gave the maximum fluorescence intensity in pH 9.00 buffer (Figure 3D). Due to L-cysteine, the surface of the nanoclusters has negatively charged in the acidic region. In this media, the fluorescence intensities were low because of the neutralization of the negatively charged CuNCs by protons.

On the other hand, the highest fluorescence emission intensity was obtained at pH 9. This may be due to the change in surface charge of CuNCs depending on pH. Sunset yellow was found to be stable at all pHs. Therefore, pH 9 was chosen as the experimental condition.

The reaction of CuNCs with SY between 0 and 30 min was examined and no significant change was after 1 min. Therefore, the reaction time was determined as 1 minute for this method to be developed and tested on different samples (Figure S1).

### 3.4. Selectivity of method

The purpose of this study is to determine the amount of SY in different types of powdered drinks. Therefore, to investigate the selectivity of the developed sensor, the effect of substances that may interfere in powder drinks such as sucrose, glucose, ascorbic acid, citric acid, tartaric acid, and the effect of ions such as Na^+^, K^+^, Mg^2+^, Ca^2+^, Cl^−^, SO_4_^2−^ were investigated. The concentration of potentially interfering substances was prepared 30 times the concentration of SY. Then, the changes in the fluorescence intensity of CuNCs were investigated by using solutions of SY and other substances that might interfere. Even if the solutions of potentially interfering substances were prepared 30 times more concentrated, CuNCs and SY gave a high (F_0_–F)/F_0_ value (Table 1). In other words, the developed method is significantly selective for SY.

### 3.5. Fluorescence quenching mechanism

**Figure 4:**
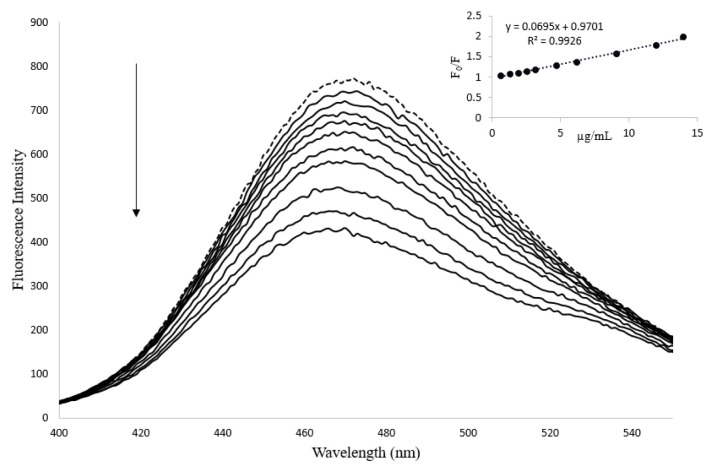
Change in fluorescent emission of CuNCs with increasing SY concentration. SY concentration is 0.65–14 μg/mL in pH 9 borate buffer (The dotted line is CuNCs alone).

L-cysteine-coated CuNCs were chosen as fluorescent probes due to their high emission, high stability, low toxicity, and easy synthesis procedure. The surface of L-cysteine functionalized CuNCs have a carboxylic acid group. SY has sulfonic groups. Both the oxygen on the carboxylic acid and on the sulfonic acid group are electronegative, hence, a new complex may form between dye and nanoclusters due to interaction among of them. As seen in Figure 4, the fluorescence emission of CuNCs quenches regularly against increasing SY concentration. The remarkable quenching of the fluorescence emission of CuNCs indicates significant interaction with SY. A blue shift was observed in the emission wavelength of CuNCs, indicating that the CuNCs changed in size.

Fluorescent quenching mechanism is of two types as dynamic (collisional) and static quenching. In the static system, the quencher and the fluorophore interact at the ground state to form a nonfluorescent complex, while in the dynamic quenching process, the molecules are in contact in the excited state and nonradiative relaxation happens. Static and dynamic quenching can be differentiated by half-life measurements of both mechanisms and their response to temperature and viscosity differences [21].

Another common quenching mechanism is the inner filter effect. For this mechanism to occur, the absorption spectrum of the quencher must overlap the excitation or emission spectrum of the donor. These mentioned mechanisms can occur simultaneously and competitively.

The quenching mechanisms can be defined Stern-Volmer equation, F_0/_F = 1 + K_SV_ [Q]. In this equation F_0_ and F are described as the fluorescence intensity of CuNCs alone and after the addition of SY, respectively. K_SV_ and [Q] represent the Stern-Volmer quenching constant and SY concentration, respectively (Lakowicz, 2006). K_sv_ is calculated from the slope of the relationship between F_0_/F and [Q]. In this study, linearity (F_0_/F = 3.1 × 10^4^ [Q] + 0.9703) was achieved in the concentration range of 1.4 × 10 ^−6^ –31 × 10 ^−6^ M with a regression coefficient of r^2^ = 0.9930 at 298 K. Obtained high linearity results explained that fluorescence quenching mechanism static and K_SV_ was calculated to be 3.1 × 10^4^ M ^−1^ which means strong interaction between the molecules.

In order to better evaluate the quenching mechanism, the temperature effect on the interaction between SY and CuNCs was studied. As seen in Table 2, Ksv values were calculated to be 4.9 × 10^4^, 3.1 × 10^4^, and 2.9 × 10^4^ at 288, 298, 308 K, respectively. K_sv_ values decreased as the temperature increased, which indicated that the possible quenching mechanism between CuNCs and SY was static [22].

Using the equation log ((F_0_–F)/F) = log K + n log [Q], binding coefficient (K) and the number of binding sites (n) can be calculated. The K value indicates whether the intermolecular interaction is strong or weak, while the n value indicates how many points there is interaction. These values are determined from the intercept and slope values of the regression curve of log(F_0_ –F)/F versus log[Q], respectively. K and n values are found as 7.2 × 10^4^ and 1.08, respectively, which means SY and CuNCs bind strongly and from a single site.

Figure 2A shows the overlapping of the absorption spectra of CuNCs and SY. In addition, there was a wide part of overlap between the emission and excitation band of CuNCs and the absorption peak of SY, which means the absorption of the excitation wavelength of CuNCs by SY/reabsorption of the NCs emission by SY. In accordance with Beer–Lambert Law, the molar absorption coefficient of SY was calculated as 4468.96, and 15786.2 at 375, and 470 nm wavelength respectively, which demonstrated that it was applicable to sensitive determination by IFE. According to these results, it can be said that the quenching mechanism is based on static and IFE. Considering the overlap of absorption spectra of dye and nanoclusters and the experimental data on the effect of temperature on quenching, it was thought that the quenching mechanism between SY and CuNCs may be a combination of static quenching and inner filter effect.

### 3.6. Fluorescence detection of SY by CuNCs

SY is used as a food dye, in candies, powder drinks, pharmaceuticals, and colorant in cosmetics. Its low cost and high stability make it more attractive and therefore more used. However, the use of high amounts has various harms, these are known as cancer, suppression of the immune system, and damage to various organs. For this reason, its use is prohibited in Finland and Norway. Therefore, the determination of SY becomes important. The interaction of SY with CuNCs causes quenching of fluorescence emission and decreasing of emission intensity depends on the concentration of the SY. This showed that the developed method is suitable to be used as a probe for the determination of SY in samples.

The proposed fluorescent sensor was studied by adding different concentrations of SY by spectrometric titration method under optimum conditions. The emission fluorescence intensity of CuNCs was measured at 470 nm when excited at 375 nm. As shown in Figure 4, the emission intensity of the nanoparticles gradually decreased as a result of the interaction with the dye. The linearity of this developed nanosensor was obtained in the range of 0.65–14 μg.mL^−1^ SY concentrations (Table 3). The R^2^ value was found to be 0.9926, so a good linear correlation was obtained between the SY concentration and the intensity. The linear regression equation ΔP = 0.0695 C + 0.97, where C is the concentration of SY (μg.mL^−1^), and ΔP is the fluorescence quenching intensity (F_0_/F). The limit of detection (LOD) and limit of quantification (LOQ) were calculated by using the 3s/m and 10s/m, where s is standard deviation and m is the slope of calibration graph, respectively [23], respectively. These values were found as 0.10 μg.mL^−1^ and 0.35 μg.mL^−1^, respectively (Table 3). Based on these results, it has been understood that the developed nanosensor is reliable, applicable and has enough sensitivity.

The repeatability of the developed method was investigated by measuring the fluorescence intensity three times on the same day (intra-day precision) and three times on followed three different days (interday precision). The percentage relative standard deviation (RSD%) was found to be 0.20 for intra-days and 0.27 for interdays (Table 3). The fact that the percent relative standard deviation is extremely low indicates that the developed method is repeatable.

According to ICH guideline Q2(R2) on the validation of analytical procedures, accuracy is usually verified by one of the following studies; comparison with reference product, spiking study, and comparison with standard method [24]. Accuracy is reported here as the average percent recovery by assaying a known amount of standard analyte added to the sample. In order to test the accuracy and reproducibility of the developed method, the recovery values were calculated by adding the stock SY solution to four different powder drink samples. Recovery values were found to be within acceptable limits between 90.3% and 105.8%. SY amounts in powder drink samples were found between 10.6–15.6 g.kg^−1^ (Table 4).

The acceptable daily intake of SY is 4 mg/kg according to European Food Safety Authority (EFSA). Even if a healthy person finishes a pack in a day, it does not exceed EFSA limits. The developed method has sufficient sensitivity to determine the allowable amount limits. When the method we developed is compared with the literatures about the determination of SY with different techniques based on chromatographic electrochemical, and fluorometric techniques, which are very sensitive methods, it is seen that proposed method has a wide linear range, and low LOD and LOQ values (Table 5). In addition, the method has advantages such as simplicity, not requiring expert personnel, using basic equipment in laboratories, and being environmentally friendly.

## 4. Conclusion

SY has undesirable harmful effects due to its use. Although cancer comes first among these effects, there are other unwanted effects such as suppression of the immune system and hyperactivity disorder. Due to these effects of SY, it is necessary to develop sensitive, reproducible, easy, and inexpensive methods.

A new, specific, and easy method was developed for the determination of SY in powder drinks using CuNCs as fluorescent probes. L-cysteine was used as the reducing and stabilizing agent for the formation of CuNCs. L-cysteine was shown to bind to the surface of CuNCs from the -SH group. In this method, there was quenching in the emission intensity of CuNCs, which was linear with increasing SY concentration. This linear reduction was tested between 0.65–14 μg.ml^−1^ SY concentrations. The Stern-Volmer equation was used to explain the quenching mechanism and it was understood that the quenching mechanism was based on static and IFE (Inner filter effect). The LOD value of 0.1 μg.ml^−1^ indicates that it can compete with other studies in the literature. Compared to other methods, this method is simple, low-cost, fast, reproducible, sensitive, and environmentally friendly.

## Supplementary file

Figure S1 Time optimization of CuNCs-SY interaction.

## Figures and Tables

**Table 1:** Effect of various interfering substances.

Sample	Added	(F_0_–F)/F ± SD
Sunset Yellow	0.013 mM	1.0 ± 8.8 × 10^−3^
Citric acid	0.39 mM	0.07 ± 0.03
Glucose	0.39 mM	0.10 ± 4.26 × 10^−3^
Sucrose	0.39 mM	0.04 ± 2.0 × 10^−3^
Tartaric acid	0.39 mM	0.052 ± 4.65 × 10^−3^
Sodium	0.39 mM	0.04 ± 5.55 × 10^−3^
Potassium	0.39 mM	0.05 ± 4.10 × 10^−3^
Magnesium	0.39 mM	0.04 ± 3.4 × 10^−3^
Calcium	0.39 mM	0.01 ± 4.20 × 10^−3^
Chloride	0.39 mM	0.04 ± 5.55 × 10^−3^
Sulfate	0.39 mM	0.04 ± 3.4 × 10^−3^

* Mean of the three experiments.

**Table 2:** Thermodynamic parameters of CuNCs-SY at three temperatures (288, 298, and 308 K).

Temperature (K)	K_sv_	n	R^2^
288	4.9 × 10^4^	0.97	0.9958
298	3.1 × 10^4^	0.97	0.9930
308	2.9 × 10^4^	1.05	0.9924

**Table 3:** Statistical evaluation of calibration data of SY using CuNCs as fluorescence probes.

Linearity range (μg.mL^−^^1^)	0.65–14
Slope	6.95 × 10^−2^
Intercept	0.97
Correlation coefficient	0.9926
SE of slope	3.6 × 10^−3^
SE of intercept	4.48 × 10^−3^
LOD (μg.mL^−1^)	0.10
LOQ (μg.mL^−1^)	0.35
Intra-day precision* (RSD%)	0.20
Inter-day precision* (RSD%)	0.27

SE is the standard error, and RSD is the relative standard deviation.

* Mean of the three experiments.

**Table 4:** Results of powder drink samples and recovery analysis of SY.

Sample	Sample value (g.kg^−1^)	Added (μg.mL ^− 1^)	Found (μg.mL^−1^) ± SD	RSD (%)	Recovery (%)
Powder drink I	15.6	2.5	2.39 ± 9.15 x 10^−3^	0.38	95.9
		5	5.04 ± 0.055	1.10	100.8
		10	10.58 ± 0.035	0.33	105.8
Powder drink II	12.1	2.5	2.34 ± 0.04	1.70	93.5
		5	4.83 ± 0.03	0.62	96.66
		10	10.15 ± 0.05	0.49	101.5
Powder drink III	9	2.5	2.26 ± 0.06	2.65	90.3
		5	4.63 ± 7 x 10^−3^	0.15	92.5
		10	10.26 ± 0.12	1.17	102.6
Powder drink IV	10.6	2.5	2.27 ± 5 x 10^−3^	0.22	90.8
		5	4.54 ± 7 x 10^−3^	0.15	90.8
		10	9.97 ± 0.112	1.12	99.7

* Mean of the three experiments.

**Table 5:** Comparison of the method developed for SY with different techniques in the literature.

Method	Linear Range(μg.mL^−1^)	LOD (μg.mL^−1^)	Recovery (%)	Reference
RP-HPLC	0.75–10	0.25	–	[25]
Electrochemical	8.6–122	2.6	80–120	[26]
Electrochemical	1.81–27.1	1.07	89–115	[27]
Electrochemical	0.5–1696	0.15	95.5–103.1	[28]
Chemiluminescence	0.15–11.0	0.1	95.45–104.62	[29]
Fluorescence	0.22–22.6/0.9–22.6	0.05/0.2	96.3–103.8	[30]
Fluorescence	0.11–27.14	0.09	99.8–103.6	[31]
This study	0.65–14	0.1	90.3–105.8	-

## References

[1] Tajik S Beitollahi H Hydrothermal synthesis of CuFe2O4 nanoparticles for highly sensitive electrochemical detection of sunset yellow Food and Chemical Toxicology 2022 165 113048 10.1016/j.fct.2022.113048 35523384

[2] Li T Ma X Xue G Ju X Liu J Determination of sunset yellow in beverage based on solution-gated graphene transistors with multi-walled carbon nanotube functionalized gate electrodes Journal of Electroanalytical Chemistry 2022 922 116758 10.1016/j.jelechem.2022.116758

[3] Li L Zheng H Guo L Qu L Yu L Construction of novel electrochemical sensors based on bimetallic nanoparticle functionalized graphene for determination of sunset yellow in soft drink Journal of Electroanalytical Chemistry 2019 833 393 400 10.1016/j.jelechem.2018.11.059

[4] Calam TT Çakıcı GT Optimization of square wave voltammetry parameters by response surface methodology for the determination of Sunset yellow using an electrochemical sensor based on Purpald® Food Chemistry 2023 404 134412 10.1016/j.foodchem.2022.134412 36228479

[5] Ma K Jiang XX Zhao M Cai YQ Simultaneous determination of 20 food additives in drinks by high performance liquid chromatography coupled with photo-diode array detector Chinese Journal of Analytical Chemistry 2012 40 1661 1667 10.3724/sp.j.1096.2012.20176

[6] El Shahawi MS Hamza A Al Sibaai AA Bashammakh AS Al Saidi HM A new method for analysis of sunset yellow in food samples based on cloud point extraction prior to spectrophotometric determination Journal of Industrial and Engineering Chemistry 2013 19 2 529 535 10.1016/j.jiec.2012.09.008

[7] Huang HY Shih YC Chen YC Determining eight colorants in milk beverages by capillary electrophoresis Journal of Chromatography A 2002 959 1–2 317 325 10.1016/S0021-9673(02)00441-7 12141558

[8] Xu L Yang F Dias AC Zhang X Development of quantum dot-linked immunosorbent assay (QLISA) and ELISA for the detection of sunset yellow in foods and beverages Food Chemistry 2022 385 132648 10.1016/j.foodchem.2022.132648 35278733

[9] Demirhan BE Kara HEŞ Demirhan B One-step green aqueous synthesis of blue light emitting copper nanoclusters for quantitative determination of food color Ponceau 4R Journal of Photochemistry and Photobiology A: Chemistry 2021 417 113356 10.1016/j.jphotochem.2021.113356

[10] Bilkay M Kara HEŞ Fluorometric determination of ornidazole by using BSA coated copper nanoclusters as a novel turn off sensor Turkish Journal of Chemistry 2022 46 2 475 486 10.55730/1300-0527.3321 PMC10734750 38143469

[11] Zhang L Wang E Metal nanoclusters: new fluorescent probes for sensors and bioimaging Nano Today 2014 9 1 132 157 10.1016/j.nantod.2014.02.010

[12] Li S Li G Shi H Yang M Tan W A fluorescent probe based on tryptophan-coated silver nanoclusters for copper (II) ions detection and bioimaging in cells Microchemical Journal 2022 175 107222 10.1016/j.microc.2022.107222

[13] Nabi AG Hussain A Di Tommaso D Ab initio random structure searching and catalytic properties of copper-based nanocluster with Earth-abundant metals for the electrocatalytic CO2-to-CO conversion Molecular Catalysis 2022 527 112406 10.1016/j.mcat.2022.112406

[14] Aparna RS Syamchand SS George S Tannic acid stabilised copper nanocluster developed through microwave mediated synthesis as a fluorescent probe for the turn on detection of dopamine Journal of Cluster Science 2017 28 2223 2238 10.1007/s10876-017-1221-1

[15] Kang J Gao P Zhang G Shi L Zhou Y Rapid sonochemical synthesis of copper nanoclusters with red fluorescence for highly sensitive detection of silver ions Microchemical Journal 2022 178 107370 10.1016/j.microc.2022.107370

[16] Sheng J Chen S Zhang J Li J Yu J UV-light irradiation induced copper nanoclusters in a silicate glass International Journal of Hydrogen Energy 2009 34 2 1119 1122 10.1016/j.ijhydene.2008.10.063

[17] Lu Y Wei W Chen W Copper nanoclusters: Synthesis, characterization and properties Chinese Science Bulletin 2012 57 41 47 10.1007/s11434-011-4896-y

[18] Mukhija A Kishore N Thermodynamic insights into interaction of protein coated gold nanoclusters with DNA and influence of coating on drug binding Journal of Molecular Liquids 2019 283 558 572 10.1016/j.molliq.2019.03.117

[19] Luo Y Miao H Yang X Glutathione-stabilized Cu nanoclusters as fluorescent probes for sensing pH and vitamin B1 Talanta 2015 144 488 495 10.1016/j.talanta.2015.07.001 26452852

[20] Ling Y Li JX Qu F Li NB Luo HQ Rapid fluorescence assay for Sudan dyes using polyethyleneimine-coated copper nanoclusters Microchimica Acta 2014 181 1069 1075 10.1007/s00604-014-1214-9

[21] Lakowicz JR Principles of fluorescence spectroscopy Boston, MA springer US 2006 10.1007/978-0-387-46312-4_2

[22] Zhang G Fu P Wang L Hu M Molecular spectroscopic studies of farrerol interaction with calf thymus DNA Journal of agricultural and food chemistry 2011 59 16 8944 8952 10.1021/jf2019006 21761894

[23] ICH Q2(R1) Validation of Analytical Procedures Text and Methodology Guidance for Industry 2005

[24] ICH Q2(R2) Guideline on validation of analytical procedures 2022

[25] Alp H Başkan D Yaşar A Yaylı N Ocak Ü Simultaneous determination of sunset yellow FCF, allura red AC, quinoline yellow WS, and tartrazine in food samples by RP-HPLC Journal of Chemistry 2018 2018 10.1155/2018/6486250

[26] Khanfar MF Abu Nameh ES Al Azizi N Zurayk RA Khalaf A Electrochemical Determination of Sunset Yellow and Tartrazine at Carbon Electrodes Modified by Fe-Zr Oxide Jordan Journal of Chemistry (JJC) 2020 15 3 119 126 10.47014/15.3.3

[27] Alves GF de Faria LV Lisboa TP de Souza CC Fernandes BLM A portable and affordable paper electrochemical platform for the simultaneous detection of sunset yellow and tartrazine in food beverages and desserts Microchemical Journal 2022 181 107799 10.1016/j.microc.2022.107799

[28] Kamalabadi M Razavi Mashouf MM Madrakian T Ghoorchian A Afkhami A Electrochemically controlled solid phase microextraction based on nanostructured polypyrrole film for selective extraction of sunset yellow in food samples Journal of the Iranian Chemical Society 2021 18 11 3127 3135 10.1007/s13738-021-02259-z

[29] Hassan MA Abdullah NS Hassan RO Majidi B Use of flow injection analysis with chemiluminescence detection for determination of sunset yellow (E110) in drink samples Journal of the Iranian Chemical Society 2020 17 6 1369 1375 10.1007/s13738-020-01859-5

[30] Wu S Zhang Y Yang L Li CP Label-Free Fluorescent Determination of Sunset Yellow in Soft Drinks Based on an Indicator-Displacement Assay Journal of Food Quality 2018 2018 1 9 10.1155/2018/6302345

[31] Kaur A Gupta U Hasan I Muhammad R Khan RA Synthesis of highly fluorescent carbon dots from spices for determination of sunset yellow in beverages Microchemical Journal 2021 170 106720 10.1016/j.microc.2021.106720

