# Supplementary figures and images for: Development of a new nanosensor for the determination of food coloring Sunset Yellow in powder drinks using L-cysteine coated copper nanoclusters
Source: Turk J Chem. 2024 Jan 18;48(2):218–28. doi: 10.55730/1300-0527.3654 (PMC11265852; doi:10.55730/1300-0527.3654)

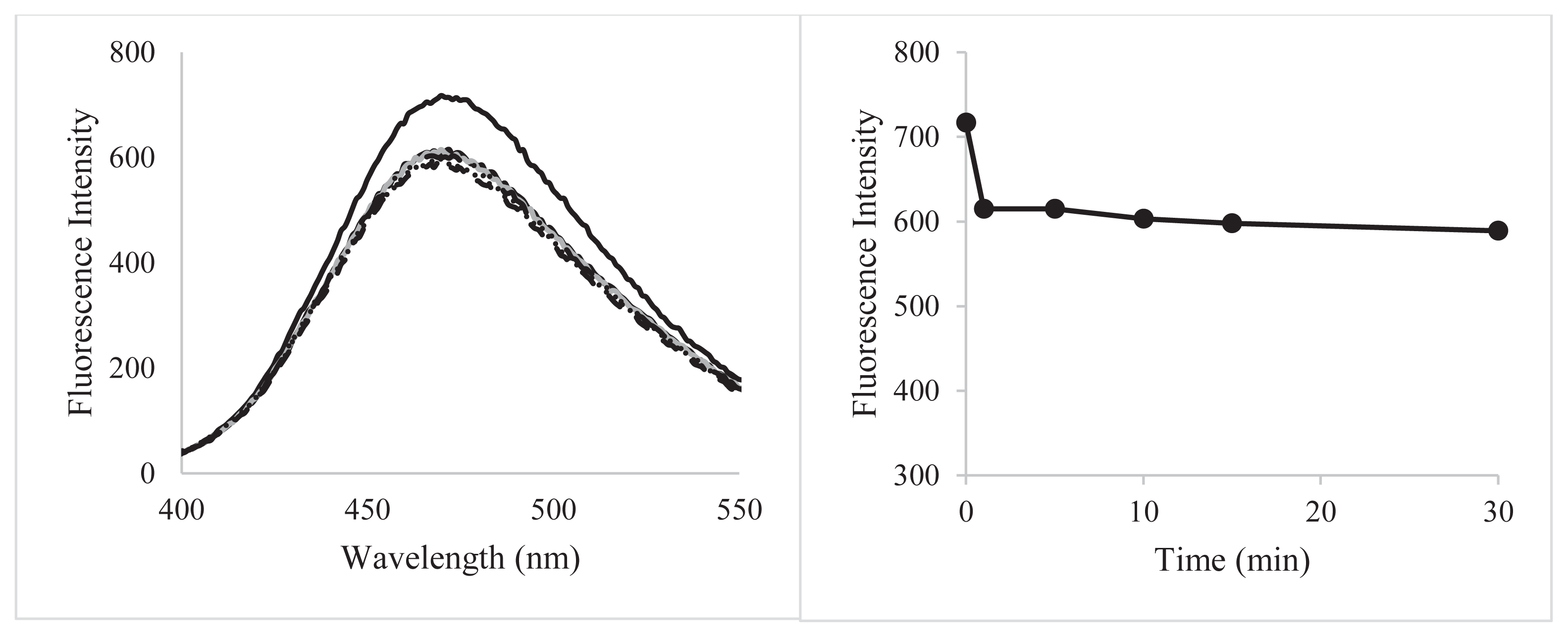

Supplement: Figure S1 — Time optimization of CuNCs-SY interaction. [file tjc-48-02-218s1.tif]
